# Activity-friendly environments for active aging: The physical, social, and technology environments

**DOI:** 10.3389/fpubh.2022.1080148

**Published:** 2023-01-11

**Authors:** Erja Portegijs, Chanam Lee, Xuemei Zhu

**Affiliations:** ^1^Center of Human Movement Sciences, University Medical Center Groningen, University of Groningen, Groningen, Netherlands; ^2^Department of Landscape Architecture and Urban Planning, Texas A&M University, College Station, TX, United States; ^3^Department of Architecture, Texas A&M University, College Station, TX, United States

**Keywords:** active aging, physical environment, social environment, technology environment, activity-friendly community

## Introduction

Leading an active life, both physically and socially, is crucial to maintain health and wellbeing in old age. As defined by the World Health Organization (WHO), “active aging” is “*the process of optimizing opportunities for health, participation, and security in order to enhance the quality of life as people age*” (1). It was first used as a policy concept to steer communities, cities, and countries toward aging-friendly actions, emphasizing an active approach to life and the need for older people to continue their involvement in a range of physical and social activities across different functional domains (2). But this concept was not fully operationalized considering the diverse perspectives of older individuals. To address this limitation, researchers have further developed approaches to measure and understand active aging, which reflect how an older person's actual behavioral decisions are a consequence of balancing one's capacity to move, one's opportunities to participate, and one's goals and preferences toward meaningful activities (3, 4).

Physical environments play a significant role in active aging and may facilitate or hinder opportunities for health, participation, and security. Older people's preference for “aging in place,” or the opportunity to continue residing in their current home and community for as long as possible, remains strong (5), further highlighting the need for environments that support active aging. An “activity-friendly environment” should include support from the physical environment as well as socio-cultural and community structures to enable and motivate active aging (6). In 2007, WHO proposed a framework for age-friendly cities containing eight interrelated domains covering the physical, socio-cultural, technological, and service environments (7). Despite its popularity as an original and comprehensive framework, it does not fully consider the heterogeneity of older adults and equity issues, and has limited applications/implications in middle-to-low-income countries (8–11).

In recent years, technology has become increasingly important in our lives, while enabling an active and engaged life in different ways than before. Emerging technologies also offer new tools and methods for researchers to assess both environments and behaviors as related to aging, thus enabling a better understanding of the complex person-environment interactions. However, the digital divide may be most pressing for older age groups, who may not be digitally literate or do not have the means to utilize technologies (12).

We provide expert opinions on how the physical aspects of activity-friendly environments can optimize opportunities for health, participation, and security, and thereby facilitate active aging, and how those are closely intertwined with the social and technology domains of environments. Both housing and neighborhood environments are discussed, and so are emerging trends, methods, and approaches in research and practice related to design for active aging.

## Physical environments

### The housing environment

Housing is essential for active aging, serving as not only a shelter but also a place of purpose and identity (13). As people age and develop functional limitations, the lack of person-environment fit (3, 14, 15) is often first reflected in housing environments, leading to the need for home modifications or relocation to a different home or a long-term care facility. The types and options of housing available to older people vary by location, and significant gaps often exist between the demand and supply of appropriate housing options.

An individual's housing choice is affected by multiple factors such as availability, housing policies, socio-cultural norms, and personal factors (e.g., finance, health status, and preferences). For example, physical barriers in residential units (e.g., multiple floors with stairs), prevalent in European cities, and traditional single-family zoning, common in the U.S., often force older people to move away from their familiar communities in pursuit of more suitable housing despite their preference for aging-in-place. Spatial segregation of homes suitable for different life stages limits housing options for older adults (16), compromises their opportunities to stay engaged in their communities, and marginalizes intergenerational contacts (9). Recently, in the U.S., some states (e.g., Oregon and California) and municipalities (e.g., City of Minneapolis) have initiated zoning reforms to allow multiple housing types (e.g., apartments, accessory dwelling units) to coexist in the same community (17).

Environmental attributes of senior housing (individual homes or congregate living) can affect seniors' physical activity, social engagement, independence, mobility, security, and aging-in-place. Relevant housing-level factors range from room features to the overall building layout and site plan. Examples include accessibility, assistive features (e.g., handrails), daylighting, window view, indoor-outdoor connections, transitional areas, greeneries, hallways or footpaths for walking, and destinations in or around buildings that can encourage physical/social activities (18–21).

Overall, policy interventions and innovative designs are needed to provide new housing models with more *diverse, supportive, affordable, and adaptable options within the community* that can support active aging and aging-in-place (22). This would require purposeful planning and design considerations in terms of the physical environment such as proximity to community amenities and spatial design balancing privacy and access/connection, as well as active engagement of seniors in the planning and design process to ensure the physical environment reflects their personal goals and preferences. These physical environmental features should also be integrated with supportive programs, social networks, and intelligent technologies in home services and health monitoring and management, to better support active aging (23).

### The neighborhood environment

For older adults, especially those with declining physical and cognitive resources, opportunities to participate in physical and social activities may be restricted due to the reasons beyond the individual. Features in the neighborhood environment impact older adults' ability to move about and be physically or socially active. Yet, understanding of the neighborhood as a unit has proven highly challenging. Administrative units (e.g., postal code areas, census boundaries) may not be consistent with personal perceptions of the neighborhood, which vary according to personal capacities and preferences (24, 25). Furthermore, experiences of the environment are highly individualized, as awareness of environmental features depends on exposure to the environment (where one actually uses) as well as one's individual functions (e.g., physical, sensory, and cognitive capacities) and other resources (e.g., financial resources, car availability, social support, and time restraints).

Active aging research also lacks unified definitions and standards for assessing the neighborhood environment (25). The variability of definitions and perspectives suggests the need to study person-environment relationships using multiple data sources (9, 24). Assessing one's individual capacity is a common practice in health sciences, and life-space assessments (environmental exposure) by means of a questionnaire or technologies (26) have gained popularity in the last few decades. However, accounting for multiple personal and environmental resources at once is not a common practice yet, especially not in the aging research field. Acknowledging the complexity of these relationships and the full range of factors involved, moreover, poses theoretical and methodological challenges that need to be tackled.

Despite these challenges, various environmental factors have been consistently identified as correlates of active aging in older adults. For example, physical environment features such as high street connectivity, diversity in services, sufficient infrastructure (e.g., sidewalks, trails, lighting), and availability of green spaces (e.g., parks, nature) have been found to promote physical activities, especially walking, among older adults (27). These serve as a good starting point to characterize the physical environment that supports active aging in place, to design environments conducive of active aging, and to further develop future research.

## Social environments

The growing interest in aging-in-place emphasizes the importance of positive and familiar social and physical environments as people age. Social ecological models suggest multiple domains of factors impacting health behaviors such as physical activity and emphasize added importance of social factors for older adults (28, 29). Social engagements are among the key determinants of active aging, and social environments can be either facilitators (e.g., social support, positive social networks, cohesive neighborhood) or barriers (e.g., social isolation, crime risk/exposure, social inequity) to active aging. However, mechanisms through which older adults' exposure and response to these social conditions impact active aging are not fully understood.

Researchers have pointed to the interplay between the physical and social environments (30–33). Some early evidence suggests significant roles of social places like the “Third Places” in the neighborhood, which typically include religious places, food retails and services, recreational destinations, and senior centers (34). However, more efforts are needed to identify the specific types and features of such socially-oriented places important for different groups of older adults, and how and to what extent they influence older adults' physical/social activities as well as aging-in-place. Significant heterogeneity exists across the socio-cultural and economic contexts, and therefore more context-sensitive knowledge is needed to better understand the roles and features of social environments that support active aging.

The concepts and measures of social environment used in the active aging literature are limited. Social factors can be approached from the contextual social environment perspective (e.g., neighborhood cohesion, social capital) or from the ego-centric social network perspective (e.g., size, stability, and strength of social networks/ties). While correlated with each other, these factors have distinctive roles and their specific impacts on active aging outcomes are not fully explored.

The socio-cultural environment is critical in addressing larger societal issues related to population aging, encompassing healthcare cost, caregiving burden, reduced workforce, and intergenerational conflicts. Growing efforts have been made to respond to this demographic shift from a broader policy and community level, such as the Age-Friendly Cities Framework by WHO (35) and various programs offered by Generations United and AARP in the U.S. Still, efforts are needed to better understand the physical and social environmental factors that contribute to supporting active aging across the lifespan and intergenerational interactions.

## Technology environments

Technology is increasingly implemented in home and care settings to facilitate interactions, and to support older adults' independence and participation in meaningful activities (36). Remote communication tools or smart monitoring solutions utilizing sensor-based technologies (e.g., passive infrared motion sensors, body-worn sensors, pressure sensors, video monitoring and sound recognition) may help older adults manage daily tasks (e.g., environmental reminders to initiate specific behaviors) and environmental challenges (e.g., long distance to services), and facilitate independent living (37). To prevent unequal access to these services and tools, it is crucial to develop simple and intuitive solutions. This requires participatory research in collaboration with individuals with limited digital skills and businesses willing to invest their time and resources to improve accessibility by diverse end-users including older adults (38).

Technology also helps researchers and practitioners better understand person-environment interactions. For example, Geographic Information System (GIS) is used by professionals and researchers from various fields to study georeferenced and objectively assessed features of the physical environment. In addition, understanding the subjective perceptions, experiences, and preferences of people is also crucial. Geographical mapping of participant responses can help capture subjective perceptions or experiences of the environment that vary by individuals (39, 40). Such online questionnaires including citizen science platforms enable easy data collection and are increasingly used to fulfill requirements of participatory planning in addition to or in replacement of traditional face-to-face meetings. However, without providing support to those with limited digital skills, such methods may fail to reach a large part of the older population. When the provision of technical support is feasible, such methods have been successfully used to map older adults' use of the environment and related preferences (41).

Monitoring sensors enable collection of data on aspects of behavior, capacity, and the environment from the participant's perspective, relatively passively. For example, global positioning (GPS) units can be used to map an individual's activities in the environment, generating measures of the life/activity space, locations of and distances to activity destinations, and speed and time of movement. The movement speed and its variability can reflect transportation mode (e.g., vehicle, on foot) and functional capacity (e.g., walking speed) (42, 43). Data processing and analysis of GPS data are still challenging for researchers in health sciences without advanced geospatial training (39), but methodological advancements will continue to improve user accessibility/applications.

Opportunities for research and the potential to generate new insights in person-environment interactions will stem from unique combinations of people-based (e.g., health and function) and place-based (e.g., GPS or map-based questionnaires) data collected from participants linked with existing or newly collected environmental data (e.g., GIS or audits). This implies the importance of multidisciplinary research methods and collaboration of experts from different scientific fields, such as public health, urban planning, architecture, data science, and geoinformatics. Furthermore, place-based research may generate relevant information for planners and designers, thus highlighting the relevance of involving such professional actors in research to facilitate knowledge utilization. It is also important to note that data collection, linkage, and sharing may raise ethical concerns related to privacy and bias, which should be considered carefully in both research and practice.

## Conclusion

Due to declining functions and limited energy reserves, older adults are more vulnerable to barriers in their physical and social environments than younger ones. Planning and design of activity-friendly communities for all ages should build on a deeper understanding of the complex dynamics underlying person-environment relationships considering the interlinked physical, social, and technological factors. This also suggests the need for interdisciplinary and multi-sectoral collaborations in research, intervention, and policy efforts (44). Technology will play an increasingly important role in knowledge generation as well as facilitating opportunities for active aging and aging-in-place. But it is important to ensure technological solutions are easy-to-use and accessible so that they do not present additional challenges to older adults with limited knowledge of or access to technology.

## Author contributions

All authors listed have made a substantial, direct, and intellectual contribution to the work and approved it for publication.
